# An early record of *Meloidogyne fallax* from Ireland

**DOI:** 10.3897/zookeys.643.11266

**Published:** 2017-01-05

**Authors:** Olivera Topalović, John F. Moore, Toon Janssen, Wim Bert, Gerrit Karssen

**Affiliations:** 1 National Plant Protection Organization, Wageningen, Geertjesweg 15, 6706 EA, The Netherlands; 2 Nematology Research Unit, Department of Biology, Faculty of Sciences, University of Gent, K. L. Ledeganckstraat 35, 9000 Gent, Belgium; 3 6 Maywood Lawn, Raheny, Dublin D05AD79, Ireland; 4 Julius Kühn-Institut – Federal Research Centre for Cultivated Plants (JKI), Messeweg 11-12, 38104 Braunschweig, Germany

**Keywords:** Root-knot nematode, Cork, morphology, morphometrics, host, characters

## Abstract

Root-knot nematodes, *Meloidogyne* spp., cause huge economic losses worldwide. Currently, three *Meloidogyne* spp. are present on the quarantine A2 list of EPPO, *Meloidogyne chitwoodi*, *Meloidogyne fallax* and *Meloidogyne enterolobii*. As a quarantine organism, *Meloidogyne fallax* has been detected in England and Northern Ireland on sport turf in 2011, and in England on leek in 2013. However, its presence in Ireland has probably been overlooked since 1965, when Mr. John F. Moore and Dr. Mary T. Franklin had detected a new *Meloidogyne* species for that time. While the relevant data was recorded and a preliminary manuscript describing the species was prepared but never submitted for publication, and together with the original slides, pictures and drawings, it was restudied recently. We compared the population of Irish *Meloidogyne* sp. to other similar *Meloidogyne* spp. Careful observation and comparison shows that it belongs to *Meloidogyne fallax*. The characters found to be common for Irish *Meloidogyne* sp. and *Meloidogyne fallax* are female stylet length (14.6 μm) with oval to rounded basal knobs, oval shaped perineal pattern with moderately high dorsal arch, slender stylet in males (18.5 μm) with set off and rounded basal knobs, slightly set off male head with one post-labial annule and incomplete transverse incisures, and second-stage juveniles with large and rounded stylet basal knobs, and a gradually tapering tail (46.9 μm) with a broadly rounded tip and a clearly delimitated smooth hyaline part sometimes marked by constrictions (12.9 μm). The host test and gall formation also correspond to *Meloidogyne fallax*. The identification could not be additionally supported by molecular analysis, as we were unable to extract DNA from the old permanent slides. Nevertheless, our study reveals that the *Meloidogyne* species detected in Ireland in 1965 belongs to *Meloidogyne fallax*.

## Introduction

Nematodes belonging to *Meloidogyne* spp. are among the most dangerous plant-parasitic nematodes worldwide and cause huge economic losses (Elling 2013). Out of more than 100 described *Meloidogyne* species (Hunt and Handoo 2009), three of them are present on the A2 list of EPPO at the moment, *Meloidogyne chitwoodi*, *Meloidogyne enterolobii* and *Meloidogyne fallax* (EPPO/PQR 2014). There are very few records on the presence of root-knot nematodes in Ireland. However, they attracted attention after Entwistle (2003) described the process of developing yellow patches symptoms on golf courses throughout the UK and Ireland. These samples were positive for *Meloidogyne naasi* and a new undescribed species which Karssen et al. (2004) described as *Meloidogyne minor*. In 2011, *Meloidogyne fallax* was detected on sport turf in Northern Ireland and England (EPPO 2013). There was a new record in 2013 in organic leeks (*Allium ampeloprasum* L.) in England with a very low risk to spread further. It was suspected that *Meloidogyne fallax* was introduced with plant waste and soil of leeks produced in other EU member states (EPPO, OEPP/EPPO 2013). Currently, *Meloidogyne fallax* has been declared present with a restricted distribution in Northern Ireland and England (EPPO/PQR 2014). Nevertheless, the first presence of *Meloidogyne* spp. in Ireland has been overlooked. An annual report of Plant Sciences and Crop Husbandry Division (now named Teagasc) contains information about the *Meloidogyne* species attacking tomato (Moore 1965). In December 1965, the samples of galled tomato roots from an unheated greenhouse in Clonakilty, Cork were sent to the laboratory of Horticultural & Forestry Research Centre in Kinsealy for analysis. These galls contained visible *Meloidogyne* sp. females with well developed egg masses on the root surface. After extraction from the original tomato roots and from tomato roots grown in infested soil at the laboratory, all life stages of the nematode were obtained. In addition, the annual report of the Plant Sciences and Crop Husbandry Division includes a brief description and a host range test of this species (Moore 1966). Based on observations of Mr. John F. Moore and Dr. Mary T. Franklin (Rothamsted, UK) from 1965/66, it was marked as a new species which differed from all the known species at that time based on the male head and the unique perineal pattern in females. The name of the species was proposed, in an unpublished manuscript, as *Meloidogyne corkensis*, according to the county Cork where it had been found. In 1995, the Dutch NPPO received the original material of *Meloidogyne* sp. from Cork including permanent slides of 23 whole females, 18 males, 27 second-stage juveniles and 6 female perineal patterns, an unpublished manuscript, pictures and drawings. Based on our observations of this material, we hypothesize that it belongs to *Meloidogyne fallax*, a quarantine species described five decades afterwards.

The main goal of our study was to compare the available original material of the population of *Meloidogyne* sp. detected in 1965 in Ireland to the type material of other similar *Meloidogyne* spp. Additionally, we tried to extract the DNA from the permanent slides originating from 1965 and 1966.

## Materials and methods

### Morphological and morphometrical analysis

In 1995, the Dutch NPPO received the original permanent slides of 23 whole females, 18 males, 27 second-stage juveniles and 6 female perineal patterns, including pictures, measurements and an unpublished manuscript. In 2005, all slides were re-mounted in glycerol.

Morphological observations of glycerine-embedded permanent slides of Irish *Meloidogyne* sp. were done using a compound light microscope (Zeiss Axio Imager 2). Pictures were obtained using a Leica DFC 450 digital camera. A compound light microscope (DM 2500, LEICA) equipped with differential interference contrast (DIC) was used for making drawings. Drawings and pictures were subsequently edited using GNU Image Manipulation Program (http://gimp.org). Permanent slides of the Irish *Meloidogyne* population were compared to type material (slides & living type populations) and reference populations, of similar *Meloidogyne* spp. (Table 1). See also Karssen (2002) and Karssen et al. (2004) for more details.

**Table 1.** Populations of *Meloidogyne* spp. used for comparison to the original slides of an unknown Irish species.

Species	Material	Number	Male	Female	J2
*Meloidogyne chitwoodi*	Type slides	(WT2076-WT2079)	2 paratypes	4 PP paraytpes	26 paratypes
	Reference live material	E7149	31	/	31
*Meloidogyne fallax*	Type slides	WT3127-WT3130	2 paratypes	2 PP paratypes	5 paratypes
	Type live material	E6147	30	/	30
*Meloidogyne minor*	Type slides	WT3371-WT3374	2 paratypes	2 PP paratypes	5 paratypes
	Type live material	F714-4	27	/	30
*Meloidogyne hapla*	Reference live material	C3093	/	/	23
*Meloidogyne incognita*	Reference live material	Rgi-23/42	30	/	30

### Host test

The original manuscript from 1966 describes in detail the conducted host range test of the Irish *Meloidogyne* sp.: Infested soil from the original site was placed together with a potential host plant species (seed/plant transplant) in 4-inch earthenware pots which were maintained in a glasshouse. The plant species used for the host range test are listed in Table 7. After 2 to 3 months, plants were removed from the pots and the root systems were examined for infections. The roots without visible galls were stained with cotton blue lactophenol to demonstrate if infection occurred. Infected plants are marked as a positive (+) and non-infected plants are marked as a negative (-).

## Molecular analysis

### DNA extraction

As only permanent slides of the Irish *Meloidogyne* sp. originating from 1966 were available, we attempted to extract the DNA from fixed nematodes based on Rubtsova et al. (2005). Briefly, slides of second-stage juveniles of Irish population were carefully broken with scalpel and T.A.F.-fixed specimens were transferred to staining blocks containing phosphate-buffered saline (PBS). Two protocols of DNA extraction were performed, i) with NaOH and Tween solution (Janssen et al. 2016), and ii) with Worm Lysis Buffer (20 mMTris-HCl, 100 mMKCl, 3.0 mM MgCl2, 2.0 mM DTT, 0.9% Tween 20) and Proteinase K (Rubtsova et al. 2005). As a positive control, DNA was extracted from three fresh second-stage juveniles of *Meloidogyne fallax* (source ID: E6147; host: *Lycopersicon esculentum* L.).

### PCR and gel electrophoresis

For amplification of a 120 bp region of COX1 gene, the forward primer, JB3 (5’-TTTTTTGGG CATCCTGAGGTTTAT-3’) (Bowles et al. 1992), was used in combination with a newly developed reversed primer, COIR120 (5’-ATTGGTTTTATTGGTTGTTT-3’). The 23 µl of a master mix (10x PCR buffer, 10 mM dNTPs, 0.2 µl of forward primer (10 µM), 0.2 µl of reverse primer (10 µM) and 0.06 µl of ToptaqDNA polymerase (QIAGEN)) and 2 µl of extracted DNA were used per PCR reaction. PCR conditions were 94 °C for 4 min; 4 × (94 °C for 30 sec, 58-54 °C for 30 sec (annealing T dropped 1 °C in each cycle), 72 °C for 2 min), 45 × (94 °C for 30 sec, 54 °C for 30 sec, 72 °C for 1 min); 72 °C for 10 min. Amplified PCR products were visualized using gel electrophoresis (1% agarose gel stained with GelRed (Biotium, Hayward)). The GeneRuler 250 bp DNA ladder (Thermo Fisher Scientific) was used as a reference according to the manufacturer’s instructions. The electrophoresis was run at 100V for 35 minutes. The pictures of gels were obtained after exposition to the UV light.

## Results

### Morphology

Morphological characters used for comparison in this study were selected according to Eisenback and Hirschmann (1981), Jepson (1987), Karssen (2002) and Karssen et al. (2004).

### Females

**Body shape and perineal pattern**

Females of Irish *Meloidogyne* sp. show oval to pyriform shape.

**Figure 1.**
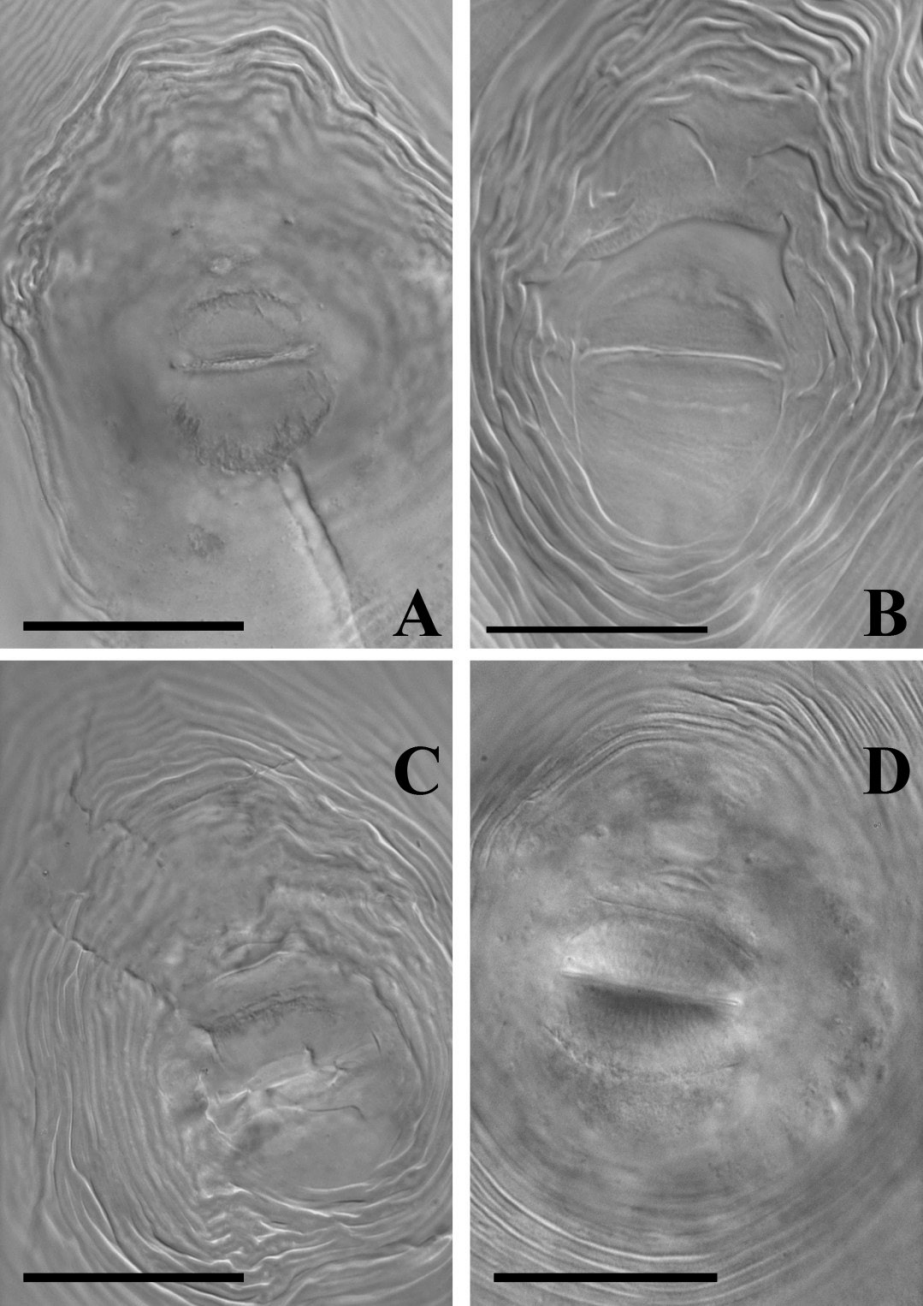
Comparison of perineal patterns in females. **A** Irish unknown *Meloidogyne* sp. **B** type material of *Meloidogyne fallax*; **C** type material of *Meloidogyne chitwoodi* **D** type material of *Meloidogyne minor*. Scale bar = 20 µm.

The perineal pattern of females of Irish *Meloidogyne* population was used for comparison according to Jepson (1987), although it is not sufficient to distinguish *Meloidogyne fallax* and *Meloidogyne chitwoodi* from each other (Karssen 2002, Karssen 1996). The perineal patterns of Irish females and type material of *Meloidogyne chitwoodi* and *Meloidogyne fallax* are similar, having ovoid to oval shape and moderately high dorsal arch. Compared to the more rounded perineal pattern in type material of *Meloidogyne minor* (Karssen et al. 2004), the correspondence of the Irish population with *Meloidogyne chitwoodi* and *Meloidogyne fallax* is more apparent (Figure 1).

**Stylet**

The stylet is slender with dorsally curved shaft. Stylet knobs are large, oval to rounded, slightly backwardly sloping, which corresponds to the original description of *Meloidogyne fallax* (Karssen 1996). Table 2 clearly shows the greatest similarity between stylet knobs of the unknown Irish species and *Meloidogyne fallax*. The stylet length, a supporting morphometrical character, is presented in Table 5 and Table 6.

**Table 2.** Differences in the stylet knob shape in females of compared *Meloidogyne* spp.

M & F + our observations	*Meloidogyne fallax* (t.l.m. + o. d.)	*Meloidogyne chitwoodi* (r.l.m. + o. d.)	*Meloidogyne minor* (t.l.m. + o. d.)	*Meloidogyne hapla* (o. d.)	*Meloidogyne incognita* (o. d.)
Large, rounded	Large, rounded	Small, irregular	Large, ovoid	Small, rounded	Large, broadly elongate

* M & F (Mr. Moore & Dr. Franklin), t. l. m. (type live material), r. l. m (reference live material), o. d. (original description)

**Second-stage juveniles**

The stylet knobs shape, tail shape and hyaline tail terminus are used for morphological observations of second-stage juveniles according to Jepson (1987) and Karssen (2002).

**Stylet knob shape**

Mr. Moore and Dr. Franklin described a slender stylet with rounded basal knobs. In specimens where it was possible to see, we observed large, rounded, set-off basal knobs that are characteristic for *Meloidogyne fallax* (see Table 3).

**Table 3.** Comparison of the most important morphological characters in second stage juveniles of the studied *Meloidogyne* spp.

	Irish *Meloidogyne* sp. (M. & F. + our observ.)	*Meloidogyne fallax* (type material + o. d.)	*Meloidogyne chitwoodi* (type slides/r. l. m. + o. d.)	*Meloidogyne minor* (type material + o. d.)	*Meloidogyne hapla* (r. l. m. + o. d.)	*Meloidogyne incognita* (r. l. m. + o. d.)
Stylet knob shape	Large, rounded	Prominent, rounded, set off	Small, irregular, sloping backwardly	Ovoid, slightly backwardly sloping	Small, rounded	Rounded, set off to transversely elongated, may indent anteriorly
Tail shape	Rounded to broadly rounded, gradually tapering until hyaline part	Gradually tapering until hyaline terminus, bluntly rounded tip	Conical, narrowly rounded tip	Gradually tapering until finely pointed tail tip, rectum weakly inflated	Short, narrow, difficult to delimitate it from hyaline region	Slightly tapering to subacute terminus
Hyaline tail terminus	Clear, rounded delimitation to the anterior, broadly rounded at the tip, sometimes with constrictions	Clearly delimitated, smooth hyaline part ending in a broadly rounded tip, faint constrictions	Short, clear rounded delimitation at the anterior end, narrowly rounded tip	Long, pointed terminus, rounded delimitation at the anterior region	Short, often irregularly shaped, delimitation at the anterior region difficult to observe	Pointed tip, clear delimitation at the anterior region

* M & F (Moore & Franklin), o. d. (original description), r. l. m. (reference live material)

**Tail shape and hyaline tail terminus**

**Figure 2.**
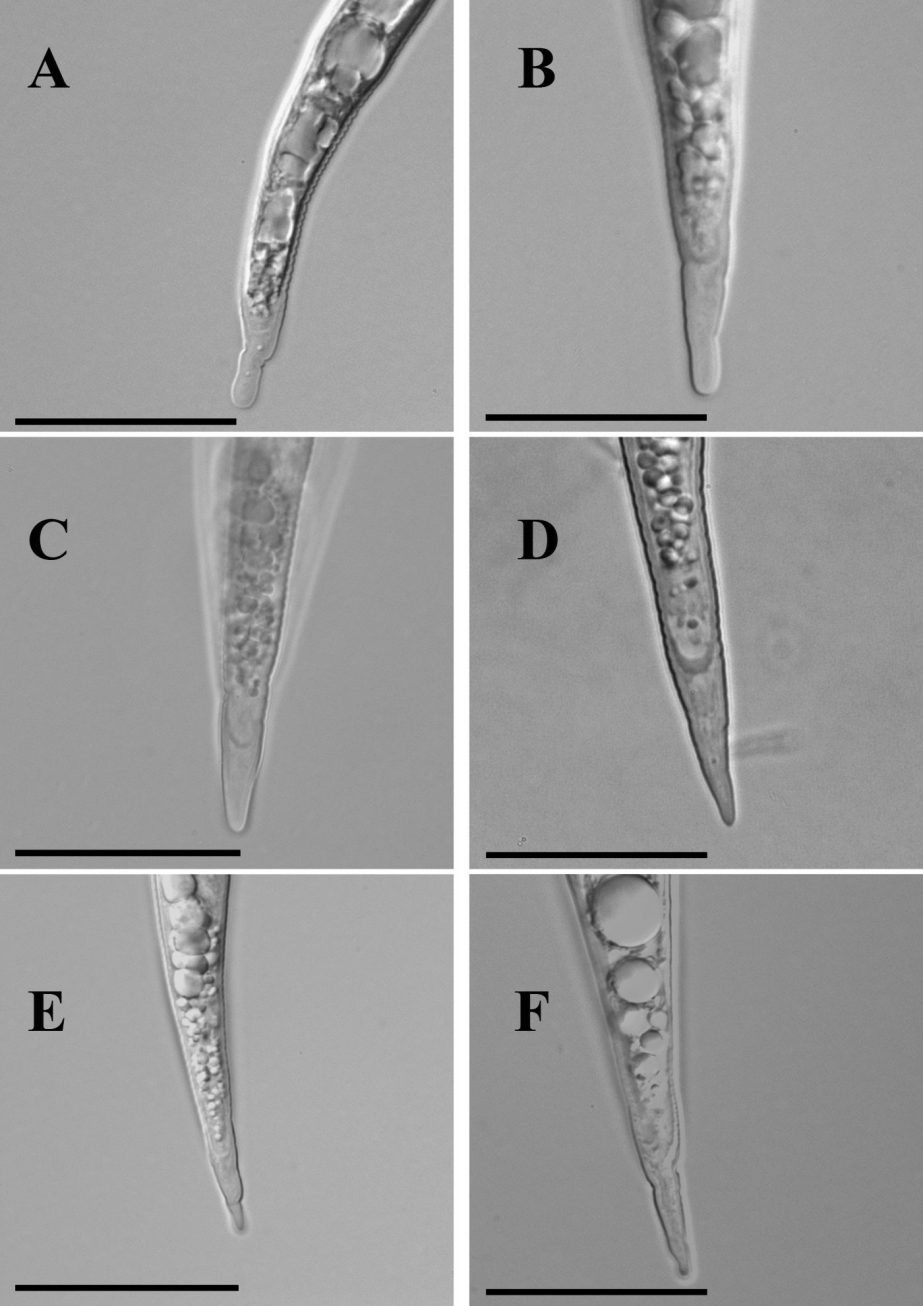
The comparison of tail and hyaline tail terminus shape in second-stage juveniles, lateral position. **A** an unknown Irish species **B** type material of *Meloidogyne fallax* **C** type material of *Meloidogyne chitwoodi* **D** type material of *Meloidogyne minor* **E** reference material of *Meloidogyne hapla*; **F** reference material of *Meloidogyne incognita*. Scale bar = 20 µm.

Mr. Moore and Dr. Franklin observed a rounded tail with a clear hyaline tail terminus which is occasionally “swollen”. Based on our observations (Table 3, Figure 2), the overall tail shape resembles the one originally described for *Meloidogyne fallax* (Karssen 1996), i.e. gradually tapering tail with a broadly rounded tip and a clearly delimitated smooth hyaline part. Some specimens in the Irish slides also show irregular constrictions at the hyaline region (Figure 5).

Our observations disagree with those of Mr. Moore and Dr. Franklin regarding the hemizonid position; it is located at the same position of the excretory pore rather than 1-2 annules above the excretory pore as they described. The position of the hemizonid at the same level of the excretory pore is characteristic for second-stage juveniles of *Meloidogyne fallax* (Karssen 1996). The hemizonid position of second-stage juveniles of other examined species is usually above the excretory pore, except for *Meloidogyne minor* where it is below the excretory pore (Karssen et al. 2004).

**Males**

Stylet knob and head shape are considered the most important characters for male identification according to Eisenback and Hirschman (1981), Jepson (1987) and Karssen (2002).

**Stylet knob shape**

**Figure 3.**
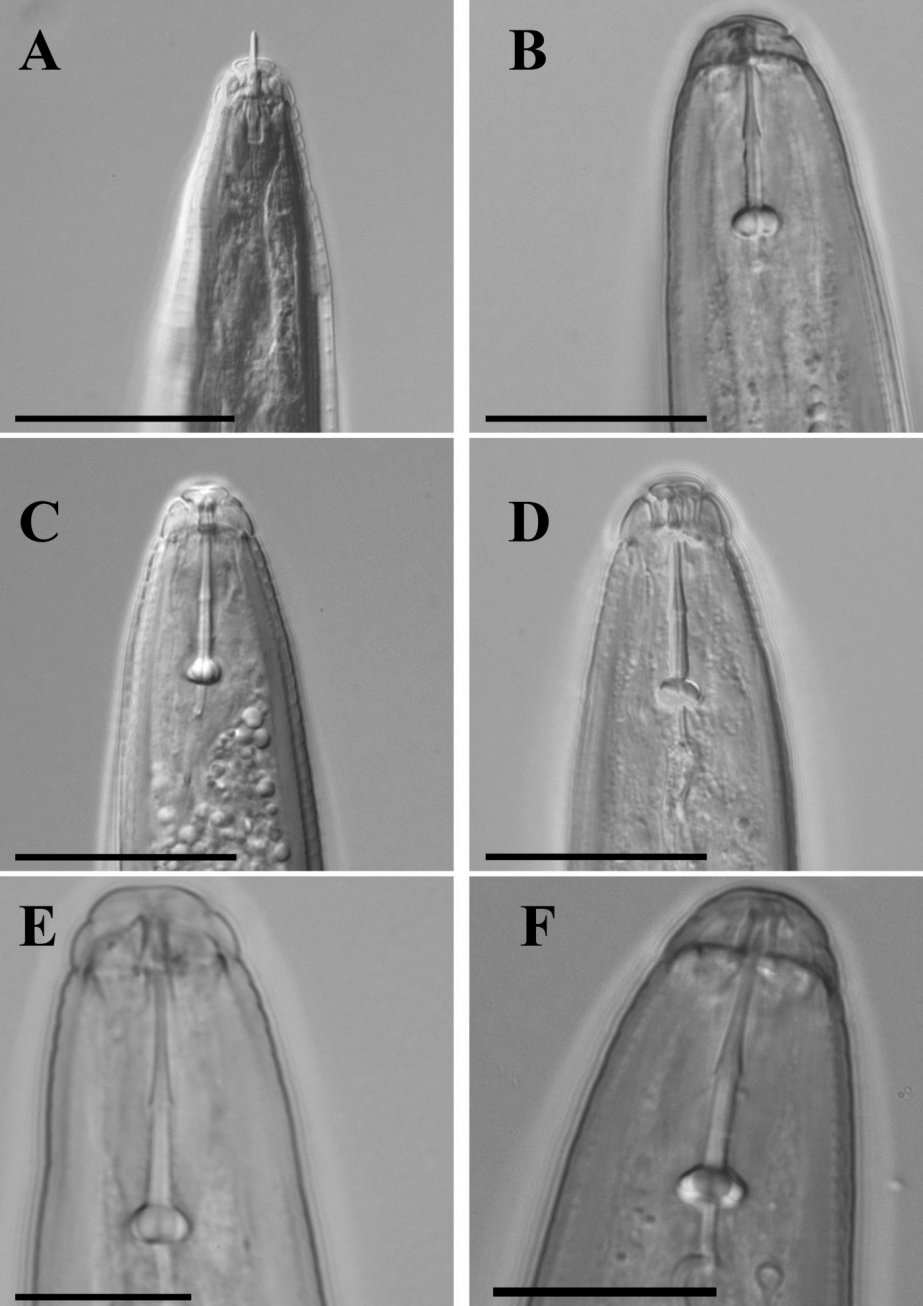
The comparison of anterior region in males of populations of observed *Meloidogyne* species. **A** an unknown Irish species (ventral position) **B** type material of *Meloidogyne fallax* (lateral position); **C**: type material of *Meloidogyne chitwoodi* (ventral position) **D** reference material of *Meloidogyne hapla* (lateral position) **E** reference material of *Meloidogyne incognita* (lateral position) **F** type material of *Meloidogyne minor* (lateral position). Scale bar = 20 µm.

As presented in Table 4, the stylet is slender with large and rounded stylet knobs, set off from the shaft, corresponding to those present in the type and reference material of *Meloidogyne fallax*. The shape of stylet knobs (Figure 3) excludes both *Meloidogyne minor*, with large, transversely ovoid stylet knobs slightly sloping backwardly (Karssen et al. 2004), and *Meloidogyne chitwoodi*, with smaller stylet knobs of irregular shape sloping backwardly (Golden et al. 1980).

**Table 4.** Stylet knob and head shape in males of compared *Meloidogyne* spp.

	M & F + our observations	*Meloidogyne fallax* (type material + o. d.)	*Meloidogyne chitwoodi* (type slides/r. l. m. + o. d.)	*Meloidogyne minor* (type material + o. d.)	*Meloidogyne hapla* (r. l. m. + o. d.)	*Meloidogyne incognita* (r. l. m. + o. d.)
Stylet knob shape	Large, rounded, set off from the shaft	Large, rounded, set off from the shaft	Smaller, oval to irregularly shaped, backwardly sloping	Larger, ovoid, slightly backwardly sloping	Small, rounded, slightly backwardly sloping	Oval, angle between the shaft and knobs is more than 90°
Head shape	Labial disc elevated, head slightly set off with a post- labial annule, sometimes with an incomplete transverse incisure, as seen from the lateral view	Labial disc rounded and elevated, head slightly set off, one post-labial annule often with an incomplete transverse incisure	Labial disc not elevated, head not set off, no transverse incisures subdividing a single post-labial annule	Labial disc elevated, head not set off, one post-labial annule often with 1-2 transverse incisures	Labial disc elevated, head swollen, no transverse incisures on a post-labial annule	Labial disc not elevated, head slightly set off, incomplete transverse incisure on a post-labial annule

**Head shape**

Mr. Moore and Dr. Franklin described three annules in lateral view of the head. The first one is deeply pinched off and succeeded by two other faintly seen annules. Our observations resemble the male head shape of type and reference material of *Meloidogyne fallax*. It is described as a slightly set off with a single post-labial annule usually subdivided with a transverse incisure (Karssen 1996). As Figure 3 shows, a labial disc is slightly elevated and typical for *Meloidogyne fallax*.

### Morphometrics

**Males**

The stylet length and stylet knob width, the most relevant morphometrical characters of males, were measured for populations of all observed species. Table 5 illustrates that the average stylet length in Irish slides is 18.5 (17.0–20.0) µm with a smaller range than previously observed by Mr. Moore and Dr. Franklin, 18.0 (15.4–24.6) µm respectively. This is similar to the average stylet length in type material (type slides and type live material) of *Meloidogyne fallax* (18.7 µm and 19.4 µm), and to type slides of *Meloidogyne minor* (18.7 µm). The average stylet knob width of 3.9 µm in Irish slides is also within the range measured for *Meloidogyne fallax* paratypes (Table 5).

**Table 5.** Morphometrical analysis of most important characters in females, males and second-stage juveniles {mean ± SD (range), all measurements in µm}.

***Meloidogyne incognita* (r. l. m.)**		/	20.2±2.1 (18.0–25.0)	4.1±0.8 (3.0–6.0)	379.2±20.0 (340.0–435.0)	55.0±2.9 (48.0–61.0)	12.2±1.7 (9.0–15.0)
***Meloidogyne hapla* (r. l. m.)**		/	/	/	364.2±31.3 (300–410)	41.1±6.8 (31.0–50.0)	8.8±1.2 (6.5–11.0)
***Meloidogyne minor***	**type slides**	/	18.7±0.7 (17.0–20.0)	4.0±0.3 (3.0–4.5)	369.0±32.5 (280–410)	52.8±4.4 (46.0–62.0)	16.9±1.6 (14.0–20.0)
	**t. l. m.**	/	17.0±0.0	4.0±0.0	347.8±17.4 (331.5–372.3)	49.0±3.3 (45.5–53.0)	13.8±1.9 (11.5–16.5)
***Meloidogyne chitwoodi***	**r. l. m.**	/	18.3±0.7 (17.0–19.0))	3.75±0.3 (3.0–4.0)	371.9±15.9 (330–400)	44.0±2.6 (40.0–49.0)	12.2±1.0 (10.0–14.0)
	**Type slides**	/	18.0±0.0	3.75±0.3 (3.5–4.0)	371.5±10.5 (350–385)	39.9±2.3 (36.0–43.5)	9.6±0.8 (8.0–11.0)
***Meloidogyne fallax***	**t. l. m.**	/	19.4±0.7 (18.0–21.0)	4.6±0.4 (3.5–5.0)	384.3±22.3 (330–420)	47.9±2.6 (41.0–54.0)	13.4±1.3 (10.5–15.0)
	**Type slides**	/	18.7±0.3 (18.5–19.0)	4.2±0.3 (4.0–4.5)	347±7.5 (340–360)	45.4±2.2 (43–49)	13.1±0.8 (12.0–14.0)
**Irish *Meloidogyne* sp. (our observ.)**		14.6±0.5 (14.0–15.0)	18.5±1.1 (17.0–20.0)	3.9±0.5 (3.0–4.5)	358.6±27.6 (280–410)	42.0±3.7 (33–50)	11.3±1.8 (8.5–15.5)
**Irish *Meloidogyne* sp. (M & F)**		18.0±2.4 (15.4–24.6)	19.5±1.5 (17.0–24.6)	/	406.1±16.1 (361.5–432.0)	46.9±2.5 (43.0–52.3)	12.9±1.8 (9.2–15.4)
**Character**		Female stylet length	Male stylet length	Male stylet knob width	J2 body length	J2 tail length	J2 hyaline tail length

**Second-stage juveniles**

The body length, tail length and hyaline tail length are considered the most reliable for morphometrical observation of second-stage juveniles. The body length range in our observations of Irish slides (280–410 µm) is narrower than observed by Mr. Moore and Dr. Franklin (361.5–432 µm) (Table 5). The tail length (46.9; 43.0–52.3 µm, noted by Mr. Moore and Dr. Franklin) is highly equivalent to that of *Meloidogyne fallax*, 49.3; 46.1–55.6 (Karssen 1996). It also matches the tail length measured in *Meloidogyne fallax* paratypes (47.9; 41.0–54.0 µm). The hyaline tail length (12.9; 9.2–15.4 µm) is slightly lower than originally described, 13.5; 12.2–15.8 µm (Karssen 1996) and when compared to the type live material of *Meloidogyne fallax* (13.4; 10.5–15.0 µm). However, clearly delimitated hyaline tail terminus ending in a broadly rounded tip and often with constrictions in Irish specimens resembles the one characteristic for *Meloidogyne fallax* (Figure 2).

**Females**

Although the female stylet length measured by Mr. Moore and Dr. Franklin is included in our study, it is considered unreliable as the length was measured from the anterior body end and not from the stylet tip. Therefore, the stylet length of the Irish *Meloidogyne* sp. was compared to the one originally described for species used for comparison in this study. Based on our measurements (Table 6), the average female stylet length (14.6 µm) corresponds to *Meloidogyne fallax* (14.5 µm).

**Host test**

**Figure 4.**
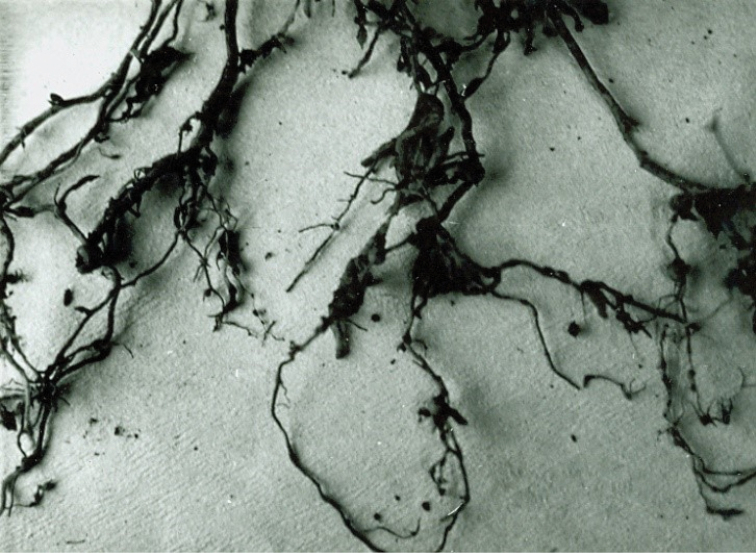
The tomato roots infected with a population of Irish *Meloidogyne* sp.

The host-range test for Irish *Meloidogyne* sp. included both weeds and cultural plants belonging to mono- and dicots. The Table 7 shows that all tested plants were positive for the infection except for *Fumaria officinalis*. The original picture from 1966 shows relatively small galls on tomato roots caused by this species (Figure 4).

**Figure 5.**
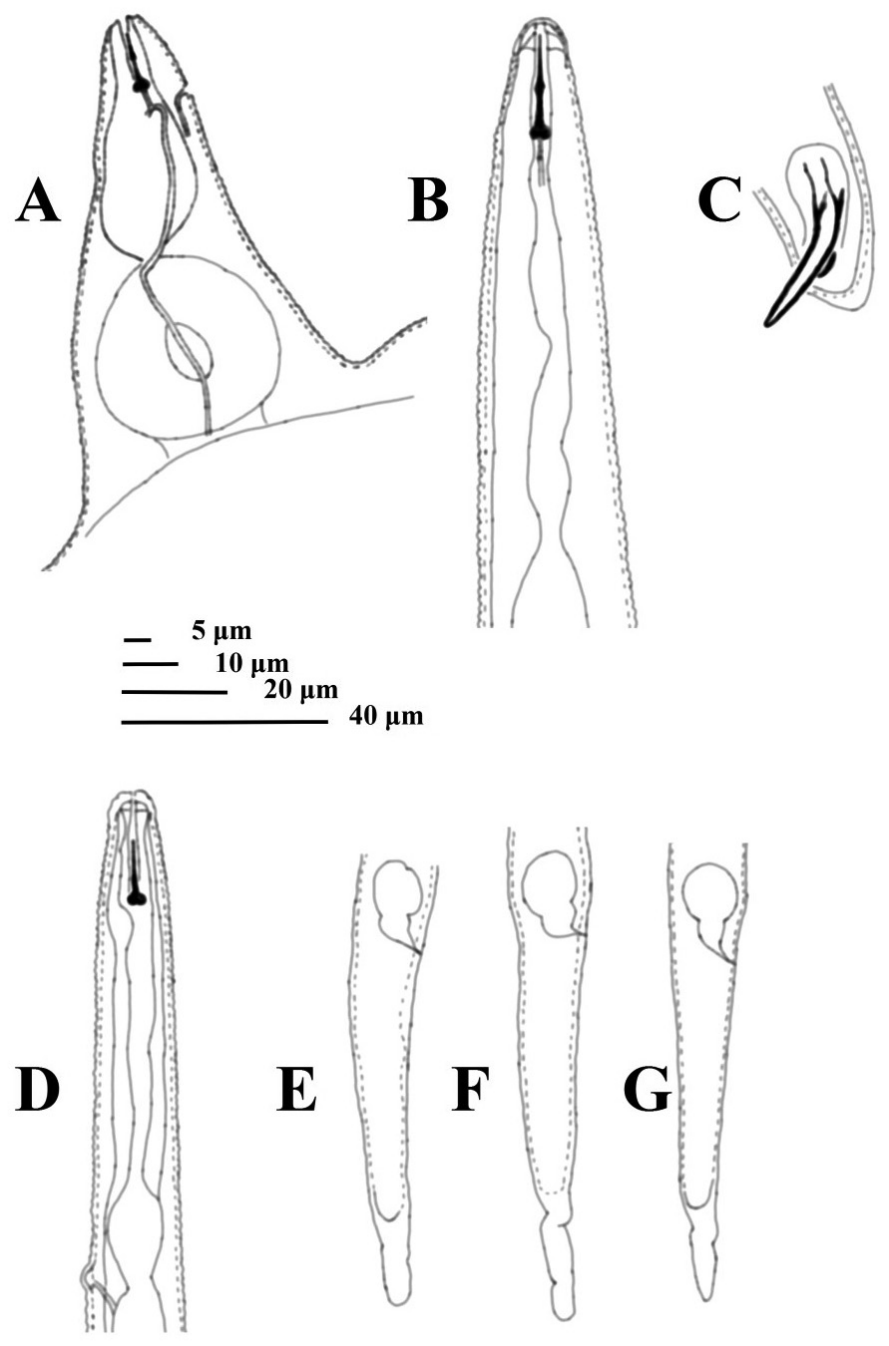
Irish population of *Meloidogyne* sp. (lateral position) from Ireland from 1965. **A** female anterior region **B** male anterior region **C** male – spicules **D** anterior region of the second-stage juvenile **E–G** tail variations in the second-stage juvenile.

**Table 6.** Comparison of female stylet length between Irish population and different *Meloidogyne* spp. {mean ± SD (range), all measurements in µm}

**Species (females)**	**Unknown Irish sp. (Moore & Franklin)**	**Unknown Irish sp. (our observations)**	***Meloidogyne fallax* (original description)**	***Meloidogyne chitwoodi* (orig. descr.)**	***Meloidogyne hapla* (orig. descr.)**	***Meloidogyne minor* (orig. descr.)**
Stylet length	18.0±2.4 (15.4–24.6)	14.6±0.5 (14.0–15.0)	14.5±0.4 (13.9–15.2)	11.9±0.3 (11.2–12.5)	13.0±/ (12.0–14.0)	14.2±1.1 (12.6–15.2)

**Molecular analysis**

The DNA extraction from glycerine-embedded nematodes in old slides was unsuccessful with both DNA extraction methods, as PCR product was not obtained. Contrastingly, the targeted region of COX1 gene was successfully amplified from all three fresh individuals of *Meloidogyne fallax*. The primers used in this study have been designed to specifically amplify a short region of COX1 gene of *Meloidogyne fallax* and *Meloidogyne chitwoodi*, the two closely related species.

## Discussion

In the annual reports of Plant Sciences and Crop Husbandry Division from 1965 and 1966, a *Meloidogyne* species attacking tomato was recorded and briefly described by Mr. John F. Moore and Dr. Mary T. Franklin. Its host range was found to be very wide, including both dicots and monocots (Table 7). Some morphological characters, such as the position of anus and vulva on a marked protuberance, the posterior cuticular pattern of females, the unique male head and different characters of the second-stage juveniles, were considered important to characterize this putative new species. The differential diagnosis was mainly made to the species belonging to the former genus *Hypsoperine* (Sledge & Golden, 1964) based on the posterior protuberance in females, although the perineal pattern was not comparable to other species. In addition, representatives of *Hypsoperine* sp., which was rejected as a valid genus (Plantard et al. 2007), were known to attack only monocots (Siddiqi 2000), while the host range of detected Irish population included both monocots and dicots.

**Table 7.** The host test conducted in 1966 for a population of Irish *Meloidogyne* sp.

Family	Genus + species	Result
Chenopodiaceae	*Beta vulgaris* L. *S	+
	*Chenopodium album* L. *Pl	+
Compositae	*Matricaria matricarioides* (Less.) Porter *Pl	+
	*Senecio jacobaea* L. *Pl	+
	*Sonchus* sp. *Pl	+
	*Lactuca sativa* L.* S	+
Caryophyllaceae	*Stellaria media* L. *Pl	+
	*Cerastium* sp. *Pl	+
Polygonaceae	*Polygonum aviculare* L. *Pl	+
	*Rumex* sp. *Pl	+
Graminaceae	*Hordeum vulgare* L. *S	+
	*Triticum aestivum* L. *S	+
	*Lolium multiflorum* (Lam.) *S	+
Cruciferae	*Capsella bursa-pastoris* L. *Pl	+
	Brassica oleracea L. var. capitata *S	+
	Brassica napus L. var. napobrassica *S	+
Euphorbiaceae	*Mercuria lisannua* L. *Pl	+
Urticaceae	*Urticadioica* L. *Pl	+
Labiatae	*Lamium purpureum* L. *Pl	+
Umbelliferae	*Daucus carota* L. *S	+
Fabaceae	*Vicia faba* L. *S	+
Plantaginaceae	*Plantago major* L. *Pl	+
Rosacae	*Fragaria vesca* L. *Pl	+
	*Potentilla erecta* L. *Pl	+
Solanaceae	*Solanum tuberosum* L. (potato tuber)	+
Ranunculaceae	*Ranunculus repens* L. *Pl	+
	*Ranunculus acris* L. *Pl	+
Geraniaceae	*Erodium moschatum* L. *Pl	+
Amaranthaceae	*Spinacea oleracea*L. *S	+
Alliaceae	*Allium cepa* L. *S	+
Papaveraceae	*Fumaria officinalis* L. *Pl	–

* S = seed sown, Pl = plant transplants sourced local to the laboratory

Our observations show that the perineal pattern of Irish females greatly corresponds to the one originally described for *Meloidogyne fallax* (Karssen 1996) and *Meloidogyne chitwoodi* (Golden et al. 1980), making it difficult to decide if the striae are more or less coarse and belong to the former or to the latter. This is why Karssen (2002) and Karssen et al. (2004) did not use perineal pattern to differentiate *Meloidogyne fallax* and *Meloidogyne chitwoodi*, even though it is considered to be one of the most important diagnostic characters by Eisenback and Hirschmann (1981) and Jepson (1987). Importantly, the stylets of some females in Irish slides had remained intact and comparison showed a high similarity to those presented in the original description of *Meloidogyne fallax* (Karssen 1996). None of measured female stylet lengths was within the range originally described for *Meloidogyne chitwoodi* (Golden et al. 1980) indicating that the two species were not mixed together. According to Mr. Moore and Dr. Franklin, the duct of dorsal pharyngeal gland opens 4-6 µm behind the stylet base. We did not mark this character as diagnostic in females following Karssen (1996) and Jepson (1987), because a physical deformation of females in permanent slides made this distance variable among different specimens.

Males and second-stage juveniles appeared to have much more informative morphological and morphometrical characters for comparison with other similar species. Mr. Moore and Dr. Franklin described the male head with 3 annules where the first one is deeply pinched off and succeeded by two faintly seen annules. Contradictory to this, we observed one post-labial annule which is interrupted with 1-2 incomplete transverse incisures visible from the lateral view on dorsal and ventral sides. We also found a slightly set-off head region with a slightly elevated labial region as was originally described for males of *Meloidogyne fallax* (Karssen 1996). To compare with, *Meloidogyne chitwoodi* males have a flattened labial region.

Our careful observations show that the stylet length of males in Irish slides matches the one measured in paratypes of *Meloidogyne fallax* and *Meloidogyne minor*. Additionally, the stylet knob shape in Irish males, being rounded and set off from the shaft as originally described for *Meloidogyne fallax*, mismatches ovoid and slightly backwardly sloping knobs characteristic for *Meloidogyne minor* (Karssen et al. 2004).

The stylet length of second-stage juveniles was excluded from the basic comparison (Table 5) as it was difficult to accurately observe the stylet tip (Jepson 1987). However, large and rounded stylet knobs set off from the shaft in Irish specimens were comparable to the ones observed in type material of *Meloidogyne fallax*, excluding both *Meloidogyne minor* and *Meloidogyne incognita* with stylet knobs slightly sloping posteriorly. On the contrary, body length of second-stage juveniles was easily observed. We noticed certain shrinkage of specimens in Irish slides compared to those observed by Mr. Moore and Dr. Franklin. This can be explained by the fact that up to 10% of shrinkage occurs after several years in slides mounted in both glycerol and lactophenol (Esser 1974). Nevertheless, the greatest correspondence of the mean body length in second-stage juveniles was found to the one described for paratypes of *Meloidogyne fallax* (Karssen 1996). In addition, Eisenback and Triantaphyllou (1991) marked body length of second-stage juveniles as inadequate for species identification due to its high overlap between different species. Jepson (1987), on the other hand, included body length as important supplementary character in root-knot nematodes identification. Remarkably, in polyploid mitotic parthenogenetic *Meloidogyne* spp., the average body length is indeed not reliable for identification as there is a high variation of this character between different individuals of the same species. Also, a large body length seems to be correlated with increased chromosome number, e.g. tetraploidic forms of *Meloidogyne microcephala* (Triantaphyllou and Hirschmann 1997) and polyploidic forms of *Meloidogyne hapla* race B compared to haploid forms of *Meloidogyne hapla* race A (Eisenback and Triantaphyllou 1991). In meiotic parthenogenetic species (e.g. *Meloidogyne fallax* and *Meloidogyne chitwoodi*) with haploid chromosome number of 18 and generally shorter body length compared to polyploidic species, high inter-specific and low intra-specific variation are sufficient enough to depict the body length as important diagnostic character.

It should also be pointed out that in Irish second-stage juveniles, a gradually tapering tail with bluntly rounded tip and a clearly delimitated hyaline part with broadly rounded tip and often constrictions resemble the tail and tail hyaline shape characteristic for *Meloidogyne fallax* (Karssen 1996). Moreover, we observed the hemizonid in second-stage juveniles positioned at the same level as the E-S pore, rejecting the observation of the hemizonid position to be 1–2 annules above the E-S pore as marked by Mr. Moore and Dr. Franklin. In fact, until Karssen (1996) described *Meloidogyne fallax*, the hemizonid had never been observed at the same level as the E-S pore. Current observation supports the taxonomic value of the hemizonid position, as it was clearly visible above the E-S pore in paratypes of *Meloidogyne chitwoodi*, *Meloidogyne hapla* and *Meloidogyne incognita*, and bellow the E-S pore in *Meloidogyne minor* paratypes.

The host test of Irish *Meloidogyne* sp. conducted in 1966 showed a wide host range which included both dicots and monocots. Although Castagnone-Sereno et al. (2013) do not consider the host test to be important for identification of certain species, we found the fact that *Meloidogyne fallax* also parasitizes monocots and dicots (EPPO 2004) as important additional argument. The original pictures of galled tomato roots made in 1965 and 1966, show relatively small galls that are typical for the roots attacked by *Meloidogyne fallax* (Karssen 1996).

An additional molecular support for our data is lacking as we were unable to extract DNA from the 50-year-old slides with both protocols used. It was confirmed by PCR amplification of COX1 gene, showing products only for fresh *Meloidogyne fallax* specimens. The COX1 gene was chosen for analysis as it has been previously proven as a good marker for distinguishing closely related *Meloidogyne* species (Humphreys-Pereira and Elling 2015). It has the advantage of being maternally inherited and linked to an actual amino acid sequence. Moreover, there are many copies of this gene in a single specimen and targeting a very short region of a multi copy gene would increase a chance for its amplification from a damaged and fragmented DNA of Irish *Meloidogyne* sp. in the old slides.

In this study we showed a historical record of *Meloidogyne fallax* in Ireland. It is not known which way it was introduced to the unheated glasshouse in the county Cork, either by infected tomato seedlings or by infested soil. Although EPPO (2004) described a direct evidence of the economic importance of *Meloidogyne fallax* as lacking and obscured compared to its sister species *Meloidogyne chitwoodi*, the fact that *Meloidogyne fallax* was present in Ireland in ’60s and again recorded in the sport turf in 2011 (Northern Ireland), indicates a continuous risk of introduction of this species in Ireland. Van der Gaag et al. (2011) relied on the matrix statistical model to assess the risk of introduction of *Meloidogyne fallax* and *Meloidogyne chitwoodi* into new countries. The outcome of this model showed a high risk of introduction of both species from France, Netherlands, Germany and UK via plant seedlings, dormant bulbs, tubers, tuberous roots, corms, crowns and rhizomes. Furthermore, a coarse soil texture in a combination with Irish climate provides good conditions for the establishment of both species.

## Conclusion

To conclude with, observations of the original material of a population of *Meloidogyne* sp. from Ireland and its comparison to other similar *Meloidogyne* spp. indicate that it belongs to *Meloidogyne fallax*.
